# Characterization of Novel Antimalarial Compound ACT-451840: Preclinical Assessment of Activity and Dose–Efficacy Modeling

**DOI:** 10.1371/journal.pmed.1002138

**Published:** 2016-10-04

**Authors:** Amélie Le Bihan, Ruben de Kanter, Iñigo Angulo-Barturen, Christoph Binkert, Christoph Boss, Reto Brun, Ralf Brunner, Stephan Buchmann, Jeremy Burrows, Koen J. Dechering, Michael Delves, Sonja Ewerling, Santiago Ferrer, Christoph Fischli, Francisco Javier Gamo–Benito, Nina F. Gnädig, Bibia Heidmann, María Belén Jiménez-Díaz, Didier Leroy, Maria Santos Martínez, Solange Meyer, Joerg J. Moehrle, Caroline L. Ng, Rintis Noviyanti, Andrea Ruecker, Laura María Sanz, Robert W. Sauerwein, Christian Scheurer, Sarah Schleiferboeck, Robert Sinden, Christopher Snyder, Judith Straimer, Grennady Wirjanata, Jutta Marfurt, Ric N. Price, Thomas Weller, Walter Fischli, David A. Fidock, Martine Clozel, Sergio Wittlin

**Affiliations:** 1 Actelion Pharmaceuticals Ltd, Allschwil, Switzerland; 2 GlaxoSmithKline, TresCantos Medicines Development Campus, Diseases of the Developing World, Tres Cantos, Madrid, Spain; 3 Swiss Tropical and Public Health Institute, Basel, Switzerland; 4 University of Basel, Basel, Switzerland; 5 Medicines for Malaria Venture, Geneva, Switzerland; 6 TropIQ Health Sciences B.V., Nijmegen, The Netherlands; 7 Department of Life Sciences, Imperial College London, SW7 2AZ, London, United Kingdom; 8 Department of Microbiology and Immunology, Columbia University Medical Center, New York, New York, United States of America; 9 Eijkman Institute for Molecular Biology, Jakarta, Indonesia; 10 Global and Tropical Health Division, Menzies School of Health Research and Charles Darwin University, Darwin, Australia; 11 Centre for Tropical Medicine and Global Health, Nuffield Department of Clinical Medicine, University of Oxford, Oxford, United Kingdom; 12 Division of Infectious Diseases, Department of Medicine, Columbia University Medical Center, New York, New York, United States of America; University of Sydney, AUSTRALIA

## Abstract

**Background:**

Artemisinin resistance observed in Southeast Asia threatens the continued use of artemisinin-based combination therapy in endemic countries. Additionally, the diversity of chemical mode of action in the global portfolio of marketed antimalarials is extremely limited. Addressing the urgent need for the development of new antimalarials, a chemical class of potent antimalarial compounds with a novel mode of action was recently identified. Herein, the preclinical characterization of one of these compounds, ACT-451840, conducted in partnership with academic and industrial groups is presented.

**Method and Findings:**

The properties of ACT-451840 are described, including its spectrum of activities against multiple life cycle stages of the human malaria parasite *Plasmodium falciparum* (asexual and sexual) and *Plasmodium vivax* (asexual) as well as oral in vivo efficacies in two murine malaria models that permit infection with the human and the rodent parasites *P*. *falciparum* and *Plasmodium berghei*, respectively. In vitro, ACT-451840 showed a 50% inhibition concentration of 0.4 nM (standard deviation [SD]: ± 0.0 nM) against the drug-sensitive *P*. *falciparum* NF54 strain. The 90% effective doses in the in vivo efficacy models were 3.7 mg/kg against *P*. *falciparum* (95% confidence interval: 3.3–4.9 mg/kg) and 13 mg/kg against *P*. *berghei* (95% confidence interval: 11–16 mg/kg). ACT-451840 potently prevented male gamete formation from the gametocyte stage with a 50% inhibition concentration of 5.89 nM (SD: ± 1.80 nM) and dose-dependently blocked oocyst development in the mosquito with a 50% inhibitory concentration of 30 nM (range: 23–39). The compound’s preclinical safety profile is presented and is in line with the published results of the first-in-man study in healthy male participants, in whom ACT-451840 was well tolerated. Pharmacokinetic/pharmacodynamic (PK/PD) modeling was applied using efficacy in the murine models (defined either as antimalarial activity or as survival) in relation to area under the concentration versus time curve (AUC), maximum observed plasma concentration (C_max_), and time above a threshold concentration. The determination of the dose–efficacy relationship of ACT-451840 under curative conditions in rodent malaria models allowed prediction of the human efficacious exposure.

**Conclusion:**

The dual activity of ACT-451840 against asexual and sexual stages of *P*. *falciparum* and the activity on *P*. *vivax* have the potential to meet the specific profile of a target compound that could replace the fast-acting artemisinin component and harbor additional gametocytocidal activity and, thereby, transmission-blocking properties. The fast parasite reduction ratio (PRR) and gametocytocidal effect of ACT-451840 were recently also confirmed in a clinical proof-of-concept (POC) study.

## Abstract 2

Sergio Wittlin and colleagues report in vivo and in vitro modelling of antimalarial activity and dose-efficacy of a new drug candidate.

## Author Summary

**Why Was This Study Done?:**

- The limited diversity of chemical mode of action in the global portfolio of marketed antimalarials along with recently observed artemisinin resistance that threatens the current first-line treatment highlights the urgent need for development of new antimalarials.
- In accordance with target product profiles defined by the Medicines for Malaria Venture ([MMV]; www.mmv.org), a new model of not-for-profit public–private partnership providing guidance to the development of new drugs, ACT-451840, a new chemical class of potent compounds with a novel mode of action, was selected for early development.
- This manuscript integrates a number of studies performed to characterize preclinically ACT-451840 and illustrates the new antimalarial drug development paradigm.

**What Did the Researchers Do and Find?:**

- This study used in vitro models to investigate compound activity on sexual and asexual blood stages and a mouse model to study the human parasite *P*. *falciparum* in vivo.
- While being fully active against artemisinin-resistant strains, ACT-451840 shares many of the favorable properties of artemisinins like its fast onset of action, activity against all asexual blood stage forms, and a PRR of >4 log per parasite cycle.
- Pharmacodynamic properties of ACT-451840 were interpreted in two mouse models of malaria with respect to the pharmacokinetic properties, with the objective to establish the portable PK/PD parameters to estimate efficacious exposure.

**What Do These Findings Mean?:**

- Modeling the dose–efficacy relationship of ACT-451840 supported prediction of the human efficacious exposure and therefore laid the groundwork for the new clinical development phases.
- Confirmed in an experimental human malaria infection model, the properties of ACT-451840, including its fast action observed in in vitro and in vivo models and its transmission-blocking activity, suggest this compound may be able to replace the artemisinin component in artemisinin-based combination therapy.

## Introduction

Malaria caused 438,000 deaths worldwide in 2015, of which 70% were in children under the age of 5 y [1]. Between 2000 and 2015, strategies for malaria control and eradication reduced the incidence of malaria by 48% in the WHO African Region. The upscaled interventions consisted of increased accessibility to long-lasting insecticidal bed nets, protection of the population at risk by indoor residual spraying, and increased access to rapid diagnostic tests and artemisinin-based combination therapies. However, with the detection of parasite resistance to artemisinin, the core compound of artemisinin-based combination therapies, in five countries of Southeast Asia, the availability of efficacious combination therapies is under threat [1]. Additionally, not a single new chemical class of antimalarials has been registered since 1996 [2], and the current global portfolio of antimalarial compounds in late clinical development relies largely on novel combinations of existing drugs, not novel compounds [3]. These elements highlight the critical and urgent need for new drugs to treat malaria.

In this search, ACT-213615, a compound from Actelion Pharmaceuticals Ltd. (ACT) with a mode of action distinct from that of all registered antimalarials, was recently described [4]. The compound was discovered in a phenotypic screen and showed potent and fast-acting activity in in vitro assays against all asexual erythrocytic stages of *P*. *falciparum* (i.e., rings, trophozoites, and schizonts). Further investigations regarding the mode of action established an interaction of this compound class with *P*. *falciparum* multidrug resistance protein-1 (PfMDR1) [5,6].

The present work illustrates a novel antimalarial drug development process, in that the studies were performed in partnership with research groups around the globe from industry and academia. MMV (Geneva, Switzerland), a new model of not-for-profit public–private partnership, was instrumental in the establishment of those collaborations and has transformed the field of malaria therapy by providing guidance to the development of new drugs. The strategy of MMV is the prioritization of new treatments that provide a single exposure radical cure and prophylaxis (SERCaP). Prevention of transmission, prevention of relapse following infection by *P*. *vivax* and *Plasmodium ovale*, and significant post-treatment prophylaxis are the key properties sought for future medicines, as described in MMV's new target compound profiles (TCP) [7]. Achievement of the SERCaP objectives might require the combination of several compounds (two to three), each with specific profiles and properties. Ideally, a new malaria treatment requires at least one molecule capable of replacing the fast-acting artemisinin component described as target compound profile 1 (TCP1). Suitability as a TCP1 candidate can be assessed in experiments (A) determining potency in both *P*. *falciparum* and *P*. *vivax* strains, (B) including strains already resistant to other antimalarials including artemisinins, (C) measuring log kill in vitro in comparison to existing antimalarials, and (D) establishing efficacy in an appropriate in vivo animal model. The TCP2-type molecules would be used as long duration combination partners that complete the clearance of the blood stage parasites not eliminated by TCP1 molecules. Understanding the PK profile of a compound will determine its suitability as a TCP2 candidate. Molecules with a potential for blocking transmission should target mature gametocytes (TCP3b) and also antagonize other nondividing parasites such as hypnozoites (TCP3a). Finally, molecules blocking the infection of the host liver by sporozoites, or killing liver schizonts, will cover the need for TCP4 drugs [8].

Leveraging these TCPs, the continuous drug discovery effort in the new class of potent antimalarials around ACT-213615 led to the selection of ACT-451840. In the present study, the characterization of ACT-451840 is used to illustrate the new drug development paradigm in which new technical expertise and model-based methodologies are being used to improve the subsequent clinical development phases and reduce attrition rates. Firstly, the experiments to evaluate the biological potential of ACT-451840 in *P*. *falciparum* in vitro assays against all asexual erythrocytic stages and against a panel of clinical isolates including *P*. *vivax* are shown. Additionally, key PD properties of the compound are described, including its precise rapidity of action and parasite log kill, efficacy in two pharmacological murine models with both human and mouse malaria parasites, and in vitro inhibition of gamete formation from the male gametocyte stage, resulting in transmission blocking in mosquitoes. Finally, the modeling of efficacy in murine models defined either as antimalarial activity, or as survival, in relation to AUC, C_max_, and time over a threshold concentration, was used to determine the dose–efficacy relationship of ACT-451840. The subsequent prediction of the human efficacious exposure was used to guide the dose selection for the initiation of the POC study. The goal of the here described preclinical assessment of activity and dose–efficacy modeling of ACT-451840 was to lay the groundwork for the next phases of clinical development.

## Methods

### Ethics Statement

Animal experiments performed at Actelion Pharmaceuticals and at the Swiss Tropical and Public Health Institute (Swiss TPH), or performed at GlaxoSmithKline (GSK) were approved, respectively, by the Swiss Cantonal Authorities and by the Diseases of the Developing World Ethical Committee on Animal Research. The animal studies carried out at GSK were in accordance with European Directive 2010/63/EU and the GSK Policy on the Care, Welfare and Treatment of Animals and were accredited by the Association for Assessment and Accreditation of Animal Laboratory Care for the ones performed at Diseases of the Developing World Laboratory Animal Science facilities. The results from the animal experiments are reported as per ARRIVE guidelines (S1 Checklist). The human biological samples were sourced ethically, and their research use was in accord with the terms of the informed consents. Ethical approval for the field studies were obtained from the Human Research Ethics Committee of the Northern Territory Department of Health & Families and Menzies School of Health Research, Darwin, Australia (HREC 2010–1396), and the Eijkman Institute Research Ethics Commission, Jakarta, Indonesia (EIREC 47).

### Synthesis of ACT-451840

The synthesis of ACT-451840 developed at Actelion Pharmaceuticals (Allschwil, Switzerland) is shown in the supporting material (S1 Fig). ACT-451840 is patented (WO 2011/083413).

### In Vitro Antimalarial Activity against *P*. *falciparum*

The in vitro antimalarial activity of ACT-451840 was measured at the Swiss TPH (Basel, Switzerland) using the [^3^H]-hypoxanthine incorporation assay [9]. Results were expressed as the concentration resulting in 50% inhibition (IC_50_). In vitro time-, stage-, and concentration-dependent effects were assessed using pyrimethamine as a stage-specific and slow-acting control, as described elsewhere [10].

### In Vitro Antimalarial Activity against *P*. *falciparum* Artemisinin-Resistant Strains

Ring-stage survival assays (RSA_0-3h_) were carried out at Columbia University Medical Center (New York, United States) as previously described [11,12]. Parasite cultures were synchronized using 5% sorbitol (Sigma-Aldrich), and schizonts were incubated in RPMI-1640 containing 15 units/ml sodium heparin for 15 min at 37°C to disrupt agglutinated erythrocytes. These schizonts were concentrated over a gradient of 75% Percoll (Sigma-Aldrich), washed once in RPMI-1640, and incubated for 3 h with fresh erythrocytes to allow time for merozoite invasion. Cultures were then subjected again to sorbitol treatment to eliminate remaining schizonts. The 0–3 h postinvasion rings were adjusted to 1% parasitemia and 2% hematocrit in 1 mL volumes (in 48-well plates) and exposed to a range of ACT-451840 drug concentration (700–35 nM) or 0.1% DMSO for 6 h. Duplicate wells were established for each parasite line ± drug concentration. One-mL cultures were then transferred to 15-mL conical tubes, centrifuged at 800xg for 5 min to pellet the cells, and the supernatants carefully removed. As a washing step to remove drug, 10 mL culture medium were added to each tube, and the cells were resuspended, centrifuged, and the medium was aspirated. Fresh medium without drug was then added to cultures, which were returned to standard culture conditions for 66 h. A media change was performed, and parasite viability was assessed by flow cytometry at 96 h. Stained parasites were detected using a BD Accuri 6 Flow Cytometer and analyzed using FlowJo Software. IC_50_ values were determined by nonlinear regression using GraphPad Prism 6.0. For statistical analyses, Student’s *t* tests were performed, and significance was set at *p* < 0.05.

### Ex Vivo Drug Susceptibility against Multidrug-Resistant *P*. *falciparum* and *P*. *vivax* Clinical Isolates

Ex vivo drug susceptibility testing in *P*. *falciparum* and *P*. *vivax* (often a mix of rings, trophozoites, and schizonts) was assessed in clinical isolates at the Timika Research Facility (Papua, Indonesia), an area with confirmed high levels of multidrug resistance to chloroquine, sulfadoxine-pyrimethamine, and amodiaquine in both species [13–15]. Thick blood films made from each well were stained with 5% Giemsa solution for 30 min and examined microscopically. The number of schizonts per 200 asexual-stage parasites was determined for each drug concentration and normalized to the control well. The dose–response data were analyzed using nonlinear regression analysis (WinNonLin 4.1, Pharsight Corporation), and the concentrations resulting in 50% inhibition (IC_50_) were derived using an inhibitory sigmoid Emax model. Ex vivo IC_50_ data were only used from predicted curves for which the E_0_ and E_max_ were within 15% of 1 and 100, respectively.

### In Vitro Parasite Reduction Ratio

In vitro PRR testing was conducted at GSK (Tres Cantos, Madrid, Spain) as previously described [16]. The assay used the limiting dilution technique to quantify the number of parasites that remained viable after drug treatment. *P*. *falciparum* strain 3D7 (MR4) was treated with drug concentration corresponding to 10 x IC_50_. Conditions of parasites exposed to treatment were identical to those used at GSK in the IC_50_ determination (2% hematocrit, 0.5% parasitemia). Parasites were treated for 120 h. Drug in culture medium was renewed daily over the entire treatment period. Parasite samples were collected from the treated culture every 24 h (24, 48, 72, 96, and 120 h time points); drug was washed out of the sample, and parasites were cultured drug-free in 96-well plates by adding fresh erythrocytes and culture medium. To quantify the number of viable parasites after treatment, 3-fold serial dilutions were used with the above mentioned samples after removing the drug. Four independent serial dilutions were performed with each sample to correct experimental variations. The number of viable parasites was determined after 21 and 28 d by counting the number of wells with growth using [^3^H]-hypoxanthine incorporation. The number of viable parasites was back-calculated by using the formula X^n-1^ where *n* is the number of wells able to render growth and X the dilution factor (when *n* = 0, number of viable parasites is estimated as zero) [16]. The PRR, defined as the logarithm to base 10 of the number of parasites the drug can kill in one parasite life cycle and indicating the killing rate of the compound investigated, was calculated as the decrease in viable parasites over 48 h. A lag phase was considered to occur for as long as drug treatment did not produce the maximal rate of killing, and this period of time was excluded for PRR calculation. The parasite clearance time to kill 99.9% of the initial population was determined using a regression calculated on the log-linear phase of the parasite reduction, taking into account the lag phase.

### In Vitro Antimalarial Activity against *P*. *berghei*

In vitro (ex vivo) activity against the mouse malaria parasite *P*. *berghei* was measured at the Swiss TPH as described elsewhere [17], with the following modifications: heparinised blood of *P*. *berghei* infected and uninfected control female NMRI mice (final hematocrit of 2.5%) was used and exposed to compounds for 16 h followed by 8 h of [^3^H]-hypoxanthine incorporation.

### In Vivo Antimalarial Efficacy Studies

In vivo efficacy against *P*. *berghei* was conducted at the Swiss TPH as previously described [18], with the modification that female NMRI mice from Harlan (Horst, The Netherlands) (*n* = 3) were infected with a GFP-transfected *P*. *berghei* ANKA strain (kindly donated by A. P. Waters and C. J. Janse, Leiden University Medical Center, The Netherlands) and parasitemia was determined using standard flow cytometry techniques. ACT-451840 was dissolved or suspended in corn oil before dosing and administered 24 h after infection (single-dose regimen) or 24, 48, and 72 h after infection (triple-dose regimen). With the single-dose regimen, blood was collected on Day 3 (72 h after infection). Samples for the triple-dose regimens were collected on Day 4 (96 h after infection). Antimalarial activity was calculated as the difference between the mean percent parasitemia for the control and treated groups expressed as a percent relative to the control group, consisting of five mice. Control mice were euthanized on Day 4 in order to prevent death typically occurring on Day 6. For the consistency in PK/PD modeling, Day 5 instead of Day 6 was considered the day of death, because treated animals were always euthanized one day before their natural death for animal welfare reasons. Animals were considered cured if there were no detectable parasites on Day 30 post-infection. To confirm the reliability of this assumption, blood from the surviving mice in curative experiments was inoculated into uninfected mice, and no parasitemia developed in these mice even after 30 d [19]. The antimalarial efficacy is defined either as antimalarial activity or as survival: the two readouts of the *P*. *berghei* murine model.

In vivo efficacy against *P*. *falciparum* was conducted at GSK according to the standard assay previously described [20,21]. Female NODscidIL2Rγnull mice were engrafted with human erythrocytes and infected with the isolate *P*. *falciparum* PfNF54^0230/N3^, a strain developed at GSK for growth in engrafted mice. At Day 3 after infection, chloroquine (at 2 or 5 mg/kg) or ACT-451840 (3, 10, 30, or 100 mg/kg; *n* = 3 mice per dose, formulated in corn oil) were dosed via oral gavage once a day for four consecutive days (quadruple-dose regimen). Blood samples were collected on Days 3, 4, 5, 6, and 7 (*n* = 3 per time point) for parasitemia measurement by flow cytometry. Similarly, blood samples were collected at 0.5, 1, 2, 4, 6, 8, 12, 24, and 48 h after a single administration of 4.7 mg/kg of ACT-451840 to a parallel group of three *P*. *falciparum-*infected mice for PK parameters determination. Results are expressed exclusively as antimalarial activity in the *P*. *falciparum* murine model.

### *P*. *falciparum* Dual Gamete Formation Assay (*Pf* DGFA)

Mature *P*. *falciparum* NF54 gametocytes were cultured at Imperial College London (London, United Kingdom), as previously described [22]. The cultures, which displayed between 0.2 and 0.4% exflagellation centers as a percentage of the total blood cell population, were then taken and divided into 96-well plates containing ACT-451840 in dose response (range 0.84 nM–20 μM). Final DMSO concentration in the assay did not exceed 0.25%. Plates were incubated for 24 h in the presence of ACT-451840 before male and female gametocyte viability was assessed by stimulating gamete formation by temperature decrease from 37°C to ~22°C and the addition of the gametocyte activating factor xanthurenic acid (5 μM). Male gamete formation was quantified by computer-aided automated identification and counting of exflagellation centers. Female gamete formation was visualized by live immunofluorescence with a Cy3-conjugated antibody specific for Pfs25—a female gamete expressed surface protein. Gamete formation was expressed as a percent inhibition, taking into consideration DMSO-negative control wells and 10 μM methylene blue-positive control wells, methylene blue being a potent inhibitor of the functional viability of male and female stage V gametocytes [22]. IC_50_ values were calculated using GraphPad Prism version 6 using the logarithmic versus normalized response (variable slope) function (Graphpad Software, San Diego, US).

### Oocyst Density Evaluation by Standard Membrane Feeding Assay

ACT-451840 was tested in the standard membrane feeding assay at TropIQ (Nijmegen, The Netherlands) in two modes, as previously described [23]. In the direct mode, the compound was directly added to a bloodmeal containing mature stage V gametocytes that was fed to *Anopheles stephensi* mosquitoes. In the indirect mode, mature stage V gametocytes were pre-exposed for 24 h to a serial dilution of compound (10^−5^ to 10^−9^ M final concentration) prior to mosquito feeding. Gametocytes were adapted to a hematocrit of 50% in full serum and fed to *A*. *stephensi* mosquitoes. Seven days after the bloodmeal, the number of oocysts in the midgut was determined by microscopy for both modes. ACT-451840 was tested in duplicate in a 9-point dose response series (10^10^–10^6^ M in 0.5 log dilutions). Vehicle control (0.1% DMSO) and positive control (10 μM dihydroartemisinin) were included. Twenty mosquitoes were dissected per sample, and oocyst load was analyzed per mosquito. All assays passed the quality control criterion of >50% infected mosquitoes in the control cages.

### Pharmacokinetic Studies

Female NMRI mice were purchased from Harlan (Horst, The Netherlands) by Actelion Pharmaceuticals. ACT-451840 was formulated as a solution in corn oil and dosed via oral gavage at 10, 100, and 300 mg/kg. Serial blood samples were collected at 0.5, 2, 4, and 8 h after dosing from two mice and after 1, 3, 6, and 24 h from two other mice, creating composite PK profiles from, in total, four mice per dose. Blood samples were analyzed using liquid chromatography coupled to tandem mass spectrometry (LC-MS/MS) after protein precipitation. PK parameters were estimated using non-compartmental analysis from the composite profiles.

### Pharmacokinetic/Pharmacodynamic Modeling

A single compartment PK model with saturable absorption was obtained using naïve pooling to fit simultaneously the observed blood concentration time data from the PK studies done at a dose of 10, 100, and 300 mg/kg, as described above. Using this model, blood concentration time profiles were predicted for all dosing regimens used in the mouse antimalarial efficacy studies. C_max_, AUC, and time above a threshold concentration were subsequently calculated using non-compartmental analysis. PD modeling was done using an E_max_ model using C_max_, AUC, or time above a threshold concentration versus survival or antimalarial activity. All modeling and PK analyses were done using Phoenix Winonlin 6.3 (Cetara, Princeton, US).

## Results

### Characterization of ACT-451840 In Vitro and In Vivo against Asexual Blood Stage: Biological Activity against Relevant Parasites

Antimalarial asexual blood-stage activity of ACT-451840 was evaluated against a panel of *P*. *falciparum* culture adapted isolates by a [_3_H] hypoxanthine incorporation assay (Table 1). ACT-451840 showed a low nanomolar activity against the drug-sensitive *P*. *falciparum* NF54 strain (mean IC_50_ = 0.4 ± 0.0 nM; IC_90_ = 0.6 ± 0.0 nM; IC_99_ = 1.2 ± 0.0 nM, *n* ≥ 3; ± SD) and was almost equally active against a number of drug-resistant isolates. The IC_50_ values of the control compounds, e.g., artesunate, chloroquine, and pyrimethamine, against the NF54 strain were 3.7 ± 0.5 nM, 11 ± 2.1 nM, and 18 ± 0.8 nM, respectively.

**Table 1:** In vitro activity against a panel of resistant and sensitive strains of *P*. *falciparum*.

Isolate (origin)	Resistance	50% inhibitory concentration (IC_50_) (nM)			
		ACT-451840	AS	CQ	PYR
NF54(West Africa)	No resistance	0.4 ± 0.0	3.7 ± 0.5	11 ± 2.1	18 ± 0.8
K1(Thailand)	CQ, SUL, PYR, CYC	0.3 ± 0.0	2.7 ± 0.4	303 ± 37	10,138 ± 705
W2(Vietnam)	CQ, SUL, PYR, CYC	0.2 ± 0.1	2.4 ± 0.7	326 ± 38	13,923 ± 3525
7G8(Brazil)	CQ, PYR, CYC	0.3 ± 0.1	1.8 ± 0.2	137 ± 21	10,484 ± 2574
TM90C2A(Thailand)	CQ, PYR, MFQ, CYC	1.0 ± 0.3	4.6 ± 1.7	174 ± 19	19,248 ± 3876
V1/S(Vietnam)	CQ, SUL, PYR, CYC	0.3 ± 0.0	3.2 ± 0.5	458 ± 66	21,936 ± 1072
D6(Sierra Leone)	MFQ	0.5 ± 0.1	7.1 ± 1.9	16 ± 1.2	5.4 ± 1.3

Abbreviations: chloroquine (CQ), pyrimethamine (PYR), cycloguanil (CYC), sulfadoxine (SUL), mefloquine (MFQ). Data are the means ± SD of *n* = 3 independent [^3^H] hypoxanthine incorporation experiments (each run in duplicate).

**Fig 1:**
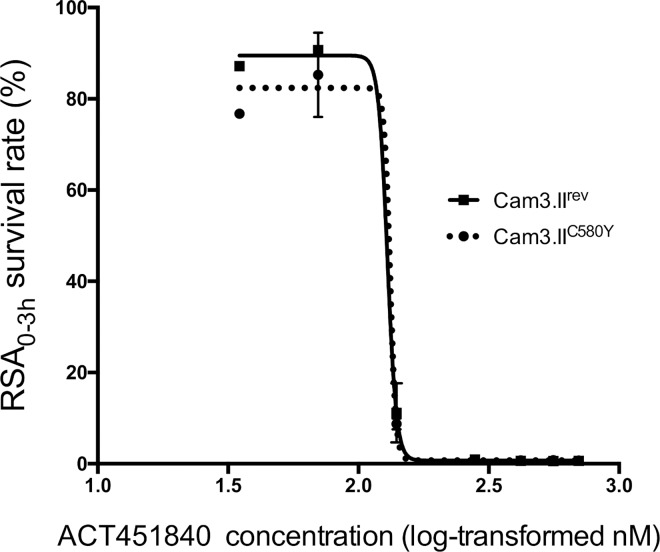
K13 propeller mutation C580Y confers no cross-resistance to ACT-451840 in ring-stage survival assays (RSA_0-3h_). Graph shows mean ± standard error (SE) ring-stage survival values in the RSA_0-3h_. At least two biological replicates were performed per line, each consisting of two technical replicates Cam3.II^C580Y^ (dotted line) and Cam3.II^rev^ (solid line). Abbreviation: ring-stage survival assay (RSA_0-3h_).

Antimalarial asexual blood-stage activity of ACT-451840 was also evaluated against a Cambodian clinical isolate (Cam3.II, which has a *K13* R539T mutation) that was gene-edited to the wild-type *K13* gene (Cam3.II^rev^) and against Cam3.II^C580Y^, a gene-edited line that is artemisinin resistant and encodes the C580Y mutation in the *K13* gene [11,12]. The mutation C580Y, conferring resistance to artemisinin, was found to not alter parasite susceptibility to ACT-451840, mefloquine, or artesunate in conventional 72-h dose–response assays that determine IC_50_ growth inhibition values on both the Cam3.II and V1/S backgrounds (S1 Text). Additionally, both Cam3.II lines were subjected to the recently developed ring-stage survival assay (RSA_0-3h_), in which parasites were exposed for a 6-h pulse to a range of drug concentration (35–700 nM), and parasite survival was assessed 66 h later [11,12]. No difference in survival rates was observed between the parasite harboring a wild-type K13 allele or the C580Y mutation (Fig 1), confirming that ACT-451840 is fully efficacious against artemisinin-resistant parasites. In contrast, the C580Y strain showed the expected grade of resistance versus the exposure to 700 nM dihydroartemisinin [11,12].

**Fig 2:**
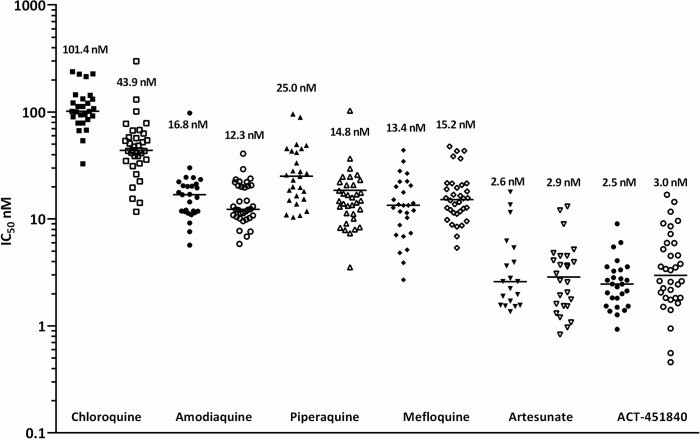
Ex vivo drug susceptibility. Median 50% inhibitory concentrations (IC_50_s) of standard antimalarials and ACT-451840 in *P*. *falciparum* (closed symbols; *n* = 27) and *P*. *vivax* (open symbols; *n* = 34) clinical isolates.

In ex vivo assays against a range of clinical isolates collected from patients with malaria from Papua, Indonesia, a region where multidrug-resistant malaria is endemic, ACT-451840 was as potent as artesunate against both *P*. *falciparum* (median IC_50_ = 2.5 [Range 0.9–9.0] nM, *n* = 27) and *P*. *vivax* (median IC_50_ = 3.0 [Range 0.5–16.8] nM, *n* = 34) (Fig 2). The IC_50_ value of ACT-451840 against the murine malaria parasite *P*. *berghei* was 13.5 nM in in vitro ex vivo assays.

**Fig 3:**
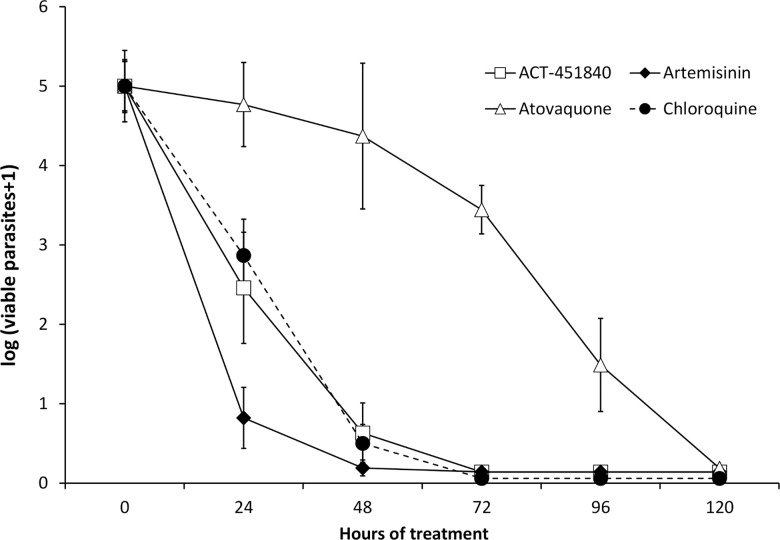
Parasite Reduction Ratio. The number of viable *P*. *falciparum* strain 3D7 (MR4) versus treatment time is compared between ACT-451840 and a selection of standard antimalarials (data for the latter was previously reported in reference [24]). Data are the mean ± SD of four independent replicates.

**Fig 4:**
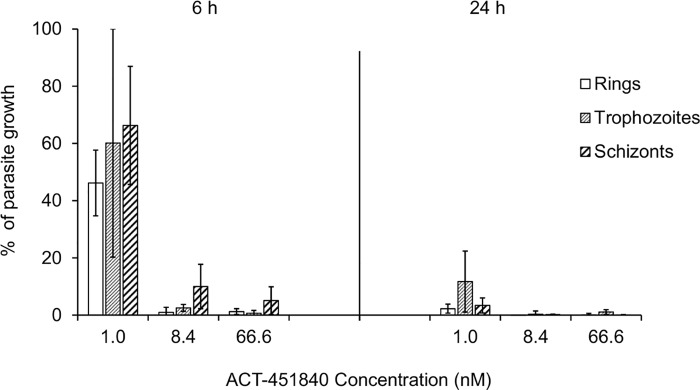
Time-, stage-, and concentration-dependent effects of ACT-451840 on synchronous cultures of *P*. *falciparum* NF54 in vitro. Parasites were exposed to ACT-451840 for 6 or 24 h at the indicated concentration. Results are expressed as the percentage of growth of the respective development stage relative to an untreated control. Each bar represents the mean + SD of three independent experiments.

In an in vitro PRR assay [16], ACT-451840 showed a fast parasite killing profile against *P*. *falciparum* similar to that of chloroquine (log PRR of 4.4 and 4.5, respectively) without lag phase in the killing curve in contrast to atovaquone (48 h). The parasite clearance time to kill 99.9% of the initial population was 28 h for ACT-451840, similar to that of artemisinin and chloroquine (less than 24 h and 32 h, respectively) (Fig 3). In vitro stage specificity assays [24] with synchronous cultures of *P*. *falciparum* NF54 were consistent with this rapid killing rate. ACT-451840 rapidly reduced parasite growth and affected all asexual erythrocytic stages equally in a time- and concentration-dependent manner, similar to artemether (Fig 4) [24].

**Fig 5:**
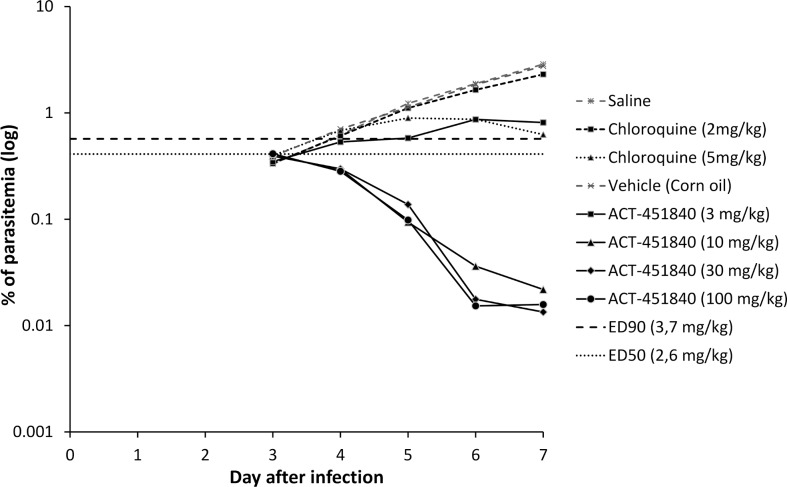
Therapeutic efficacy against *P*. *falciparum* in vivo. Parasitemia in peripheral blood of mice infected with *P*. *falciparum* NF54^0230/N3^ and treated with vehicle, chloroquine, or ACT-451840 once daily for 4 d starting on Day 3 after infection. Data shown are mean parasitemia of three mice/group. Abbreviations: 50% effective dose (ED_50_), 90% effective dose (ED_90_).

In vivo activity of ACT-451840 was assessed in the *P*. *falciparum* mouse model established at GSK (DDW, Tres Cantos, Spain). After an oral quadruple-dose regimen, ACT-451840 had a rapid onset of action and an effective dose, resulting in 90% antimalarial activity (ED_90_) of 3.7 mg/kg (3.3–4.9 mg/kg), which was comparable to the one of chloroquine (ED_90_ of 4.9 mg/kg) (Fig 5).

**Fig 6:**
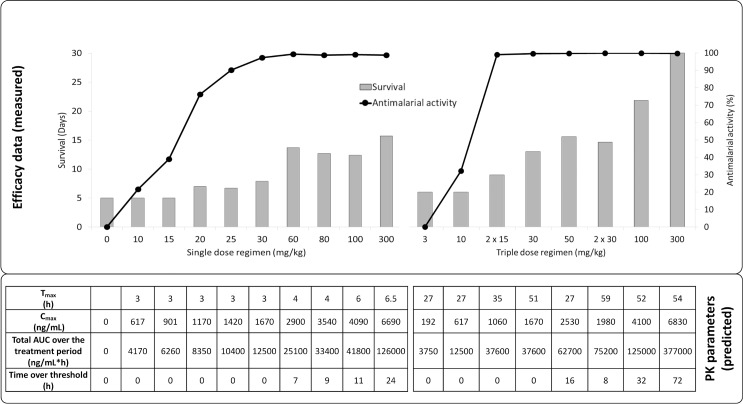
In vivo efficacy (upper panel) and exposure (lower panel) of ACT-451840 against *P*. *berghei*. Doses were administered once per day except for 2 x 15 mg/kg and 2 x 30 mg/kg, for which mice were dosed twice per day, with the second daily dose 8 h after the first dose. Abbreviations: pharmacokinetic (PK), time to reach the maximum observed plasma concentration (T_max_), maximum observed plasma concentration (C_max_), area under the concentration versus time curve (AUC).

In a *P*. *berghei* mouse model, the ED_90_ after an oral triple-dose regimen of ACT-451840 was 13 mg/kg (11–16 mg/kg) (calculated from the data in S2 Text). This ED_90_ value was about 4-fold higher than the one observed in the *P*. *falciparum* mouse model (ED_90_ 3.7 mg/kg). The difference in in vivo activities correlates with the difference in in vitro ex vivo activities of ACT-451840 against *P*. *berghei* (IC_50_ 13.5 nM, see above) and *P*. *falciparum* ex vivo (IC_50_ 2.5 nM, clinical isolates; Fig 2). Despite the lower activity of ACT-451840 against *P*. *berghei*, 20% and 100% of the infected mice were cured after 100 and 300 mg/kg triple-dose regimen, respectively (Fig 6 and S2 Text). In the same in vivo model, artesunate, the semisynthetic derivative of artemisinin, cured 50% of the mice at 100 mg/kg after a quadruple-dose regimen.

In summary, ACT-451840 had an IC_50_ below 10 nM in vitro against a panel of sensitive and resistant strains of *P*. *falciparum* and was fully active against recently identified artemisinin-resistant strains. Furthermore, the compound showed ex vivo activity against *P*. *falciparum* and *P*. *vivax* as well as a single digit mg/kg ED_90_ in a *P*. *falciparum* murine model. In addition, the compound inhibited growth of all asexual blood stages of *P*. *falciparum* in vitro, and its in vitro PRR was similar to that of chloroquine. These key properties fully meet the criteria described in TCP1 [8].

### Characterization of ACT-451840 In Vitro against Sexual Blood Stage: Potential for Blocking Transmission

Using two bioassays, the effects of ACT-451840 on the functional viability of both male and female mature gametocytes and oocysts of *P*. *falciparum* were explored to illustrate how the question of transmission is addressed.

**Fig 7:**
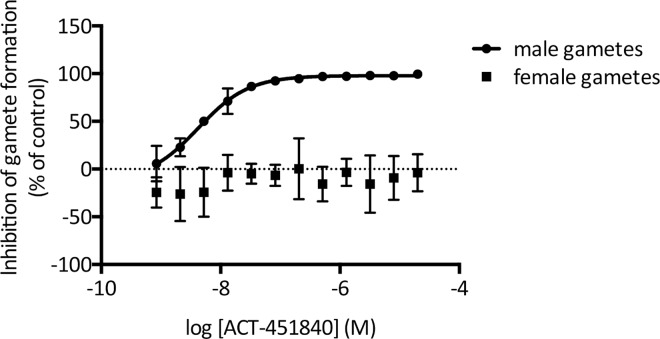
*P*. *falciparum* dual gamete formation assay. The effect of ACT-451840 on the functional viability of *P*. *falciparum* male and female gametocytes was tested in vitro in dose response. Male gametocyte viability was reported by microscopic detection of exflagellation centers and yielded an IC_50_ of 5.89 nM ± 1.80 nM (SD). ACT-451840 showed no activity against female gametocytes, as reported by the surface expression of the female gamete marker pfs-25. Data shown are the mean of four independent replicates. Error bars indicate SE.

Within the human host blood, *P*. *falciparum* gametocytes are classified morphologically into five stages, denoted I–V, whilst *P*. *vivax* gametocytes do not show the same morphology. Although the various sexual stages do not cause clinical disease, stage V gametocytes are infective to mosquitoes. These gametocytes are not eliminated by the majority of current antimalarial agents [25] and therefore remain circulating in the human bloodstream for up to 3 wk, well after the disappearance of clinical symptoms of malaria [26]. Gamete formation is a functional readout for the viability of the mature stage V gametocyte [27]. ACT-451840 potently prevented male gamete formation from the gametocyte stage with an IC_50_ of 5.89 nM ± 1.80 nM (SD), as shown in Fig 7.

Interestingly, ACT-451840 had no effect on the functional viability of female gametocytes up to a concentration of 20 μM. Nevertheless, successful transmission requires viable gametocytes of both sexes to be present, as seen similarly with two male-specific molecules, MMV007116 and MMV085203, that demonstrate transmission blocking in a standard membrane feeding assay [27], supporting the potential of ACT-451840 to block malarial transmission.

**Fig 8:**
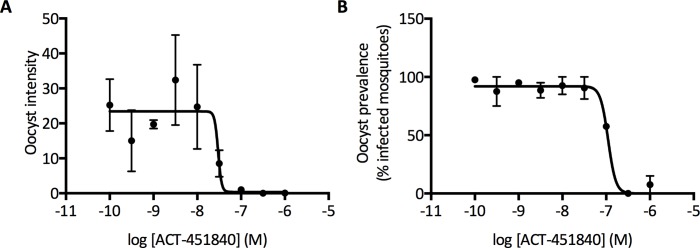
ACT-451840 blocking transmission. Standard membrane feeding assays were performed with a 24 h pre-incubation of gametocytes with compound (indirect mode). (A) shows average oocyst intensity per mosquito and (B) shows average oocyst prevalence (percentage of mosquitoes with at least one oocyst). Error bars indicate SE from the measurements of the two groups of the 20 mosquitoes per sample.

In line with this, ACT-451840 did indeed block transmission when tested in the standard membrane feeding assay. In this assay, parasite cultures containing *P*. *falciparum* stage V gametocytes were exposed to compound for 24 h prior to mosquito feeding. Seven days postinfection, control feeds showed an average parasite density of 26.2 oocysts per mosquito, which is well within the range of oocyst densities observed in laboratory infections [28]. ACT-451840 dose-dependently blocked oocyst development in the mosquito, with an IC_50_ of 30 nM (95% confidence interval: 23–39 nM) (Fig 8A). At this parasite exposure, the prevalence of infection (number of infected mosquitoes) was inhibited, with an IC_50_ of 104 nM (95% confidence interval: 98–110 nM) (Fig 8B).

As the parasite exposure in the field is much lower (1–3 oocysts/mosquito), ACT-451840 potency might be underestimated and might have a greater effect on oocyst prevalence [29]. To address the specific effect of the compound against the parasite forms that develop in the mosquito midgut, a different mode of standard membrane feeding assay was performed, in which the compound was added to the parasite at the time of mosquito feeding. Here, ACT-451840 did not show any activity against *P*. *falciparum* oocysts at concentrations up to 1 μM (S2 Fig). These data strongly suggest that the transmission-blocking effect of ACT-451840 relies on its gametocytocidal activity.

### Characterization of ACT-451840 Safety Profile: Initiation of Clinical Studies

In order to initiate investigation in humans, the preclinical safety profile of ACT-451840 was analyzed in dedicated toxicities studies performed in compliance with Good Laboratory Practice regulations and following the International Council for Harmonization guidelines. In safety pharmacology studies, no effects on central nervous and respiratory parameters were observed in rats dosed up to 1,000 mg/kg, and no effect on cardiovascular parameters were observed in dogs dosed up to 500 mg/kg. ACT-451840 was not genotoxic when assessed in vitro in a reverse mutation test or in a chromosome aberration study and in vivo in a micronucleus test. In the general oral toxicity studies performed with ACT-451840 for up to 4 wk, rats and dogs were dosed up to 2,000 mg/kg/day and 1,000 mg/kg/day, respectively. There was no evidence of local gastrointestinal toxicity. The no-observed-adverse-effect levels were established at 100 mg/kg/day in both species based on reduced body weight gain and clinical chemistry and histology findings, respectively. Based on these results, safety margins were calculated to cover the whole dose range to be tested in the single ascending dose Phase I study.

### Translation of PD and PK Data from Animal Models into Man

**Fig 9:**
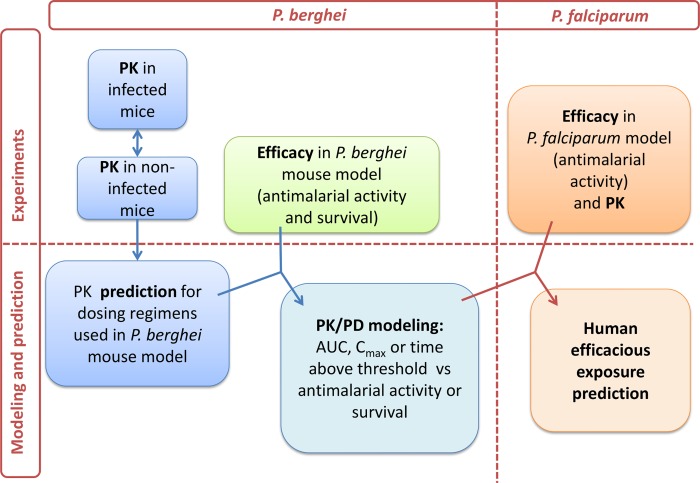
PK/PD strategy. Workflow of PK and PD modeling approach towards human efficacious dose prediction. Abbreviations: maximum observed plasma concentration (C_max_) and, area under the concentration versus time curve (AUC).

Towards the goal of initiation of a POC study and based on the results from the *P*. *falciparum* and *P*. *berghei* malaria mouse models, the human effective dose was predicted. The approach to model the PK/PD relationship is illustrated in Fig 9.

As a first step, PK parameters of ACT-451840 were determined in healthy mice after a single oral dose of 10, 100, and 300 mg/kg body weight. The PK parameters were calculated and are shown in Table 2.

**Table 2:** Pharmacokinetics parameters of ACT-451840 in healthy mice (*n* = 4).

	Pharmacokinetic parameters measured		
Dose (mg/kg)	T_max_ (h)	C_max_ (ng/mL)	AUC (ng/mL*h)
**10**	2.5	415 ± 20%	1,780 ± 18%
**100**	6	3,910 ± 20%	32,900 ± 36%
**300**	7	7,010 ± 32%	60,900 ± 33%

Abbreviations: time to reach the maximum observed plasma concentration (T_max_), maximum observed plasma concentration (C_max_), area under the concentration versus time curve (AUC).

To assess the PK parameters in infected mice, blood samples were taken at 1, 4, and 24 h after the first dose of a triple-dose regimen in the *P*. *berghei* efficacy studies. The employed doses were 15 or 30 mg/kg bis in die (b.i.d.), and 30, 50, 100, 300 mg/kg quaque die (q.d.) (*n* = 3/timepoint and dose). No significant difference in blood concentration was found between the time points studied in infected mice compared to those in healthy mice (S3 Fig).

In order to analyze the PK/PD relationship, drug exposure was predicted for all tested doses in *P*. *berghei* mouse model using a single compartment model with saturable absorption based on the concentration–time profile observed after 10, 100, and 300 mg/kg single-dose regimen in healthy mice. PK model parameter estimates are shown in Table 3.

**Table 3:** Parameterization of the pharmacokinetic model.

Parameter estimation of the mouse pharmacokinetic model	
**Volume of distribution/bioavailability (L/kg)**	4.3
**Clearance/bioavailability (L/h)**	2.4
**Max Absorption (ug/h/kg)**	21
**Concentration at half the maximal absorption (ug/mL)**	64

The PK model was used to predict the blood PK parameters for all dosing regimens used in the *P*. *berghei* mouse model, as shown in Fig 6 and S2 Text.

The next step was to determine which of the PK parameters (C_max_, AUC, or time above a threshold concentration) was driving the curative efficacy of ACT-451840 in the *P*. *berghei* mouse model. To establish this PK/PD relationship, the predicted and measured PK properties were used to model curative efficacy in relation to AUC, C_max_, and time over a threshold concentration of 1,000 ng/mL. This threshold was defined as the minimum concentration needed to significantly increase the survival of mice treated with ACT-451840 after both single- and triple-dose regimens over that of control animals. The value of 1,000 ng/mL was estimated from the data in S2 Text, in which the triple-dose regimen of 10 mg/kg with a C_max_ of 617 ng/mL showed no increase in survival compared to control animals, whereas the triple-dose regimen of 30 mg/kg with C_max_ of 1,670 ng/mL showed an increase in survival up to 13 d.

**Fig 10:**
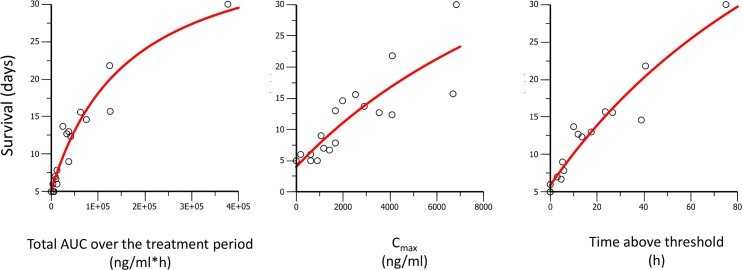
Survival days of *P*. *berghei* infected mice plotted against AUC, C_max_, and time above threshold over the entire treatment period (1 or 3 d). Dots are the observed days of survival; the line is the modeled relationship using a maximal effect (E_max_) model.

**Fig 11:**
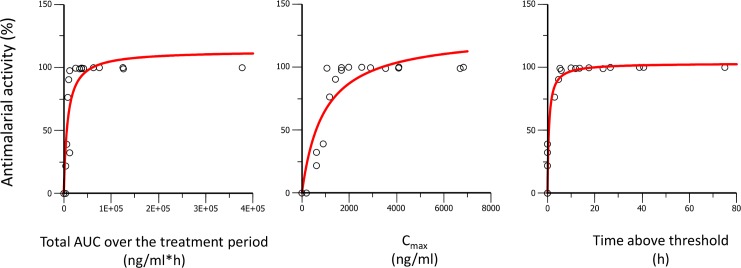
Antimalarial activity in *P*. *berghei* infected mice plotted against AUC, C_max_, and time above threshold over the entire treatment period (1 or 3 d). Dots are the observed antimalarial activity; the line is the modeled relationship using a maximal effect (E_max_) model.

The PK/PD relationships for the *P*. *berghei* in vivo efficacy experiments are given in Fig 10 and Fig 11 and the resulting PK/PD parameters are shown in Table 4.

**Table 4:** Pharmacodynamic parameters of ACT-451840 versus pharmacokinetic parameters.

	Survival			Antimalarial activity	
	EX_50_	E_0_	E_max_	EX_50_	E_max_
**AUC**	170,000 (ng*h/mL) ± 16%	5.1 (day) ± 17%	35 (day) ± 35%	8,100 (ng*h/mL) ± 35%	110 (%) ± 8.3%
**C**_**max**_	15,000 (ng/mL) ± 170%	4 (day) ± 54%	62 (day) ± 120%	930 (ng/mL) ± 36%	130 (%) ± 11%
**Time above threshold**	150 (h) ± 52%	5.9 (day) ± 12%	70 (day) ± 71%	0.14 (h) ± 3,000%	100 (%) ± 38%

Data includes coefficient of variation. E_0_ and E_max_ are the minimal effect and maximal effect, respectively, with the effect being survival or antimalarial activity. EX_50_ is the magnitude of X needed to achieve 50% of the maximal effect, with X being AUC, C_max_, or time above threshold.

The best fit for predicted versus observed PK/PD relationship was found for AUC and time above a threshold concentration versus survival, and time above a threshold concentration versus antimalarial activity, as illustrated by the correlation coefficients ≥0.96 (Table 5).

**Table 5:** Correlation of the observed survival or antimalarial activity versus modeled maximal effect relationship.

	Correlation for survival (R^2^)	Correlation for antimalarial activity (R^2^)
**AUC**	0.96	0.88
**C**_**max**_	0.85	0.91
**Time above threshold**	0.96	0.97

R^2^ = correlation between observed and predicted activity. Abbreviations: area under the concentration versus time curve (AUC), maximum observed plasma concentration (C_max_).

Survival and AUC were selected as parameters for the predictions in human. Survival is a robust readout and the relevant endpoint for clinical efficacy. AUC, rather than time above threshold, was used because the threshold value was based on PD endpoint in infected mice with *P*. *berghei* and may be different in humans infected with *P*. *falciparum*.

Having established AUC as the driver for survival in the *P*. *berghei* mouse model, this insight was used to predict an efficacious exposure against *P*. *falciparum* in humans with the results of the *P*. *falciparum* mouse model. In the *P*. *falciparum* mouse model after a quadruple-dose regimen, the ED_90_ of 3.7 mg/kg corresponded to a total AUC in blood of 227 ng*h/mL (linearly extrapolated from the exposure measured at a single dose of 4.7 mg/kg). The total AUC giving an ED_90_ in the *P*. *berghei* model (16,300 ng*h/mL after a triple-dose regimen of 13 mg/kg) was 23-fold lower than the total AUC giving cure (377,000 ng*h/mL after a triple-dose regimen of 300 mg/kg). Using this factor, the efficacious human total AUC was estimated to be 227 x 23 = 5,250 ng*h/mL, expressed in mouse blood. Correcting for the measured blood/plasma ratio of 0.6 (S3 Text), this corresponds to a predicted total efficacious human plasma AUC of 8,750 ng*h/mL. This exposure is about six times the exposure (1,408 ng*h/mL) observed after a single 500 mg dose in fed human subjects [30].

## Discussion

This report presents results from vitro and in vivo experiments to preclinically characterize a novel antimalarial compound (ACT-451840). The data shown here indicate that ACT-451840 shares many of the favorable MMV TCP1 properties of artemisinin and its derivatives, such as its potency against *P*. *falciparum* and *P*. *vivax*, fast onset of action, activity against all asexual erythrocytic stages, and PRR of >4 log per cycle of asexual blood stage forms. In addition, although the potency observed in the standard membrane feeding assay was 10-fold lower than the potency of the compound against asexual blood stage parasites, ACT-451840's gametocytocidal activity blocks transmission, fulfilling the properties requested for a TCP3b [8].

In the global portfolio of malaria medicines, other recently discovered novel compounds are the synthetic peroxide OZ439 [18], the spiroindolone KAE609 [31], the imidazolopiperazines KAF156 [32], the dihydroorotate dehydrogenase inhibitor DSM265 [33], and the translation elongation factor 2 inhibitor DDD107498 [23]. ACT-451840 shares the fast-acting properties [34] with OZ439 and KAE609, which are both foreseen to be part of a single-exposure radical cure product, as the compounds fit the TCP1, 3b and TCP1, 2 and 3b, respectively [7,35]. Although KAF156 and DDD107498 share the transmission-blocking characteristics with ACT-451840, both are seen to be agents with potential for chemoprophylaxis due to their in vitro activity against liver schizonts [23,36]. Finally, DSM265 exhibits properties of a TCP 2, positioning this compound with its long duration as potential partner of a single-exposure combination, in addition to its prophylaxis potential (TCP4) [33].

In contrast to the artemisinins, the here described compound class acts through a distinct mode of action by interacting with the *P*. *falciparum* multidrug resistance protein-1 [4,5]. Combined with the finding that ACT-451840 was well tolerated in a recent single ascending dose study and exhibited a much longer terminal half-life than artemisinin, comparable to OZ439 (34 h, 2 h, and 25–30 h, respectively) [30,37,38], this novel compound has the potential to be used as a substitute for the artemisinin derivatives in combination treatments. Additionally, the in vitro activity against artemisinin-resistant strains positions ACT-451840 as a strong candidate in the race for malaria eradication.

PD properties of ACT-451840 were interpreted in two mouse models of malaria with respect to the PK properties, with the objective to establish the portable PK/PD parameters to estimate efficacious exposure following similar methodology validated with KAE609 [35]. In this PK/PD approach, the robust endpoint of animal survival and cure were chosen for clinical relevance. Using survival as endpoint, both AUC and time above a threshold concentration were drivers for efficacy. The AUC resulting in cure in the mouse models was further translated to a predicted efficacious exposure in man, which was actually about six times the exposure observed after a single dose of 500 mg in fed state in healthy subjects. Two elements might give prospect to this conservative prediction. Firstly, the threshold concentration specified in the current PK/PD analysis (1,000 ng/mL in the *P*. *berghei* model) was defined as two times the 99% inhibitory concentration in a recent publication [35]. The alternative approach used to assess efficacious concentrations in vivo calculated the minimum inhibitory concentration and estimated a minimum parasiticidal concentration from modeling of the exposure–efficacy in the *P*. *falciparum* murine model. Because exposure was measured at a single dose of ACT-451840, this method cannot be applied in this case. However, given *P*. *berghei* and *P*. *falciparum* differences, it is likely that both concentrations would be much lower for ACT-451840 than the threshold concentration in the *P*. *berghei* model. Considering the 99% inhibitory concentration of ACT-451840, the value of the threshold would be 1.8 ng/mL (2.4 nM) and would be covered for 72 h, corresponding to one and a half asexual blood reproductive cycles, after a single dose of 500 mg in fed state in healthy subjects. Secondly, the results of the phase I study indicated the presence of circulating active metabolites with, on average, a 4-fold increase in antimalarial activity [30]. Providing these active metabolites were not present in the malaria murine model (not investigated) and, therefore, not taken into account for the human efficacious dose prediction, the overall antimalarial activity in humans dosed with ACT-451840 could be even higher than expected.

The aim of efficacious dose prediction is to provide guidance for the selection of optimal doses to be investigated in POC clinical trials as monotherapy. Although artemisinin monotherapy offers rapid recovery and fast parasite clearance [39], the use of combination therapy is recommended to reduce the risk of recrudescence associated with monotherapy, which is high for treatment regimens less than 7 d in length [40], and to decrease the potential development of drug resistance [41]. This approach allowed short-course combination therapies to be deployed with excellent efficacy, tolerability, and adherence, as illustrated with the lumefantrine/artemether combination therapy Coartem [42,43]. In addition, as fat intake enhances absorption, particularly for lumefantrine [44] and ACT-451840 [30], the development of a lipid-based formulation in humans might be the next major improvement towards limiting the variability and increasing exposure. Of note, work performed at Actelion Pharmaceuticals (S4 Text) has shown that the food effect observed with ACT-451840 [30] could be mimicked by the use of lipid-based formulations in fasted conditions in dog PK studies. Therefore, dose prediction might be refined during the development of the future medicine with new formulation and combination partners.

ACT-451840 has been tested as a monotherapy in an experimental human malaria infection model, a clinical trial developed by MMV in partnership with QIMR Berghofer of Medical Research Institute (Brisbane, Australia) [45]. The dosing regimen for this POC study (500 mg in fed condition) was constrained by the conditions tested in the phase I study. The results of this study confirmed the fast action of ACT-451840 observed in in vitro and in vivo models and its gametocytocidal effect. This validates that ACT-451840 might be a good candidate for TCP1 and TCP3b in humans [34]. The future steps will consist of the development of a lipid-based formulation, the selection of a combination partner, and the determination of the activity of ACT-451840 in patients.

## Supporting Information

S1 Checklist The ARRIVE Guidelines Checklist. (PDF)

S1 Fig Scheme of ACT-451840 synthesis. (A) **1** in MeOH, pH = 5 (AcOH), then **2** and NaBH_3_CN, 80°C, 12 h, 90%; (B) **3** in DCM, then 4M HCl in dioxane, 0°C to rt, 12 h, 63%; (C) **5** and TBTU in DCM, then DIPEA, then **4** in DCM, rt, 4 h, 93%; (D) **6** in DCM, 0°C, then 4 M HCl in dioxane, 0°C to rt, 16 h, quant. yield; (E) **8** and **9** in DMSO, then K_2_CO_3_, ultrasound, 5 h rt, then 120°C, 17h, 89%; (F) **7** and **10** in MeCN, then NaBH(OAc)_3_ in portions, rt, 16 h, 81%; (G) NaH in THF, 0°C then **13**, 0°C, 30 min, then **12**, 5 h rt, work-up and isolation of ethyl ester, used crude in (H) EtOH, KOH, 50°C, 6 h, 81% over 2 steps; (I) **14**, DCM/DMF, 20°C, then addition of (COCl)_2_ in DCM, 2 h, this solution was added to **11**, DCM/H_2_O, NaHCO_3_, 10°C, 2 h, 65%.
(TIF)

S2 Fig ACT-451840 blocking transmission. Standard membrane feeding assays were performed without a 24-h preincubation of gametocytes with compound (direct mode). The figure shows average oocyst intensity per mosquito (A) and average oocyst prevalence (percentage mosquitoes with at least one oocyst) (B). Error bars indicate SEM from the measurements of the two groups of the 20 mosquitoes per sample.
(TIF)

S3 Fig Blood-concentration time profiles in infected and healthy mice after 100mg/kg (upper panel/A) and 300 mg/kg (lower panel/B) dosing. (TIF)

S1 Text In vitro antimalarial activity against *P*. *falciparum* artemisinin-resistant strains. Antimalarial susceptibility of Cam3.II and V1/S K13 variants.
(DOCX)

S2 Text In vivo efficacy and exposure of ACT-451840 against *P*. *berghei*. (DOCX)

S3 Text Blood to plasma ratio. (DOCX)

S4 Text Effect of formulation on pharmacokinetic parameters in dogs. Pharmacokinetic parameters of ACT-451840 after 100 mg single-dose oral administration of various formulations in dogs.
(DOCX)

## Abbreviations

AUC: Area under the concentration versus time curve
B.i.d.: bis in die (bi-daily)
C_max_: Maximum observed plasma concentration
CQ: Chloroquine
CYC: Cycloguanil
DMSO: Dimethyl sulfoxide
ED_50_: 50% effective dose
ED_90_: 90% effective dose
E_0_: Minimal effect
E_max_: Maximal effect
GSK: GlaxoSmithKline
IC_50_: 50% inhibitory concentration
IC_90_: 90% inhibitory concentration
IC_99_: 99% inhibitory concentration
LC-MS/MS: Liquid chromatography coupled to tandem mass spectrometry
MFQ: Mefloquine
MMV: Medicines for Malaria Venture
MR4: Malaria Research and Reference Reagent Resource Center
NMRI: Naval Medical Research Institute
PD: Pharmacodynamics
*Pf*MDR1: *P*. *falciparum* multidrug resistance protein-1
PK: Pharmacokinetics
POC: Proof-of-concept
PRR: Parasite reduction ratio
PYR: Pyrimethamine
q.d.: Quaque die (once a day)
RPMI: Roswell Park Memorial Institute medium
RSA_0-3h_: Ring-stage survival assay
SD: Standard deviation
SE: Standard error
SERCaP: Single Exposure Radical Cure and Prophylaxis
SUL: Sulfadoxine
Swiss TPH: Swiss Tropical and Public Health Institute
TCP: Target compound profile
Tmax: Time to reach the maximum observed plasma concentration
WHO: World Health Organization
