# Supplementary figures and images for: Characterization of Novel Antimalarial Compound ACT-451840: Preclinical Assessment of Activity and Dose–Efficacy Modeling
Source: PLoS Med. 2016 Oct 4;13(10):e1002138. doi: 10.1371/journal.pmed.1002138 (PMC5049785; doi:10.1371/journal.pmed.1002138)

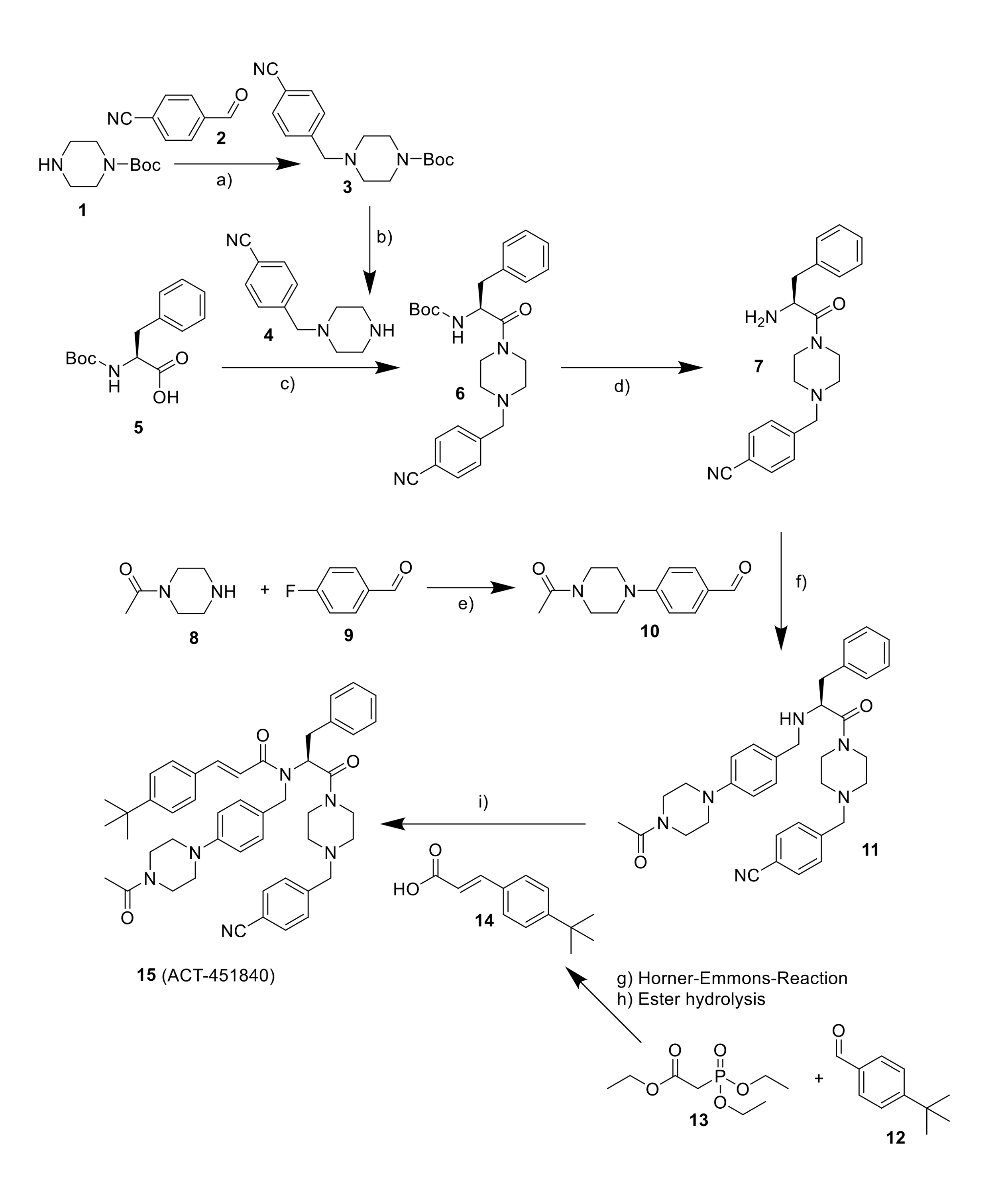

Supplement: S1 Fig — (A) 1 in MeOH, pH = 5 (AcOH), then 2 and NaBH3CN, 80°C, 12 h, 90%; (B) 3 in DCM, then 4M HCl in dioxane, 0°C to rt, 12 h, 63%; (C) 5 and TBTU in DCM, then DIPEA, then 4 in DCM, rt, 4 h, 93%; (D) 6 in DCM, 0°C, then 4 M HCl in dioxane, 0°C to rt, 16 h, quant. yield; (E) 8 and 9 in DMSO, then K2CO3, ultrasound, 5 h rt, then 120°C, 17h, 89%; (F) 7 and 10 in MeCN, then NaBH(OAc)3 in portions, rt, 16 h, 81%; (G) NaH in THF, 0°C then 13, 0°C, 30 min, then 12, 5 h rt, work-up and isolation of ethyl ester, used crude in (H) EtOH, KOH, 50°C, 6 h, 81% over 2 steps; (I) 14, DCM/DMF, 20°C, then addition of (COCl)2 in DCM, 2 h, this solution was added to 11, DCM/H2O, NaHCO3, 10°C, 2 h, 65%. (TIF) [file pmed.1002138.s002.tif]

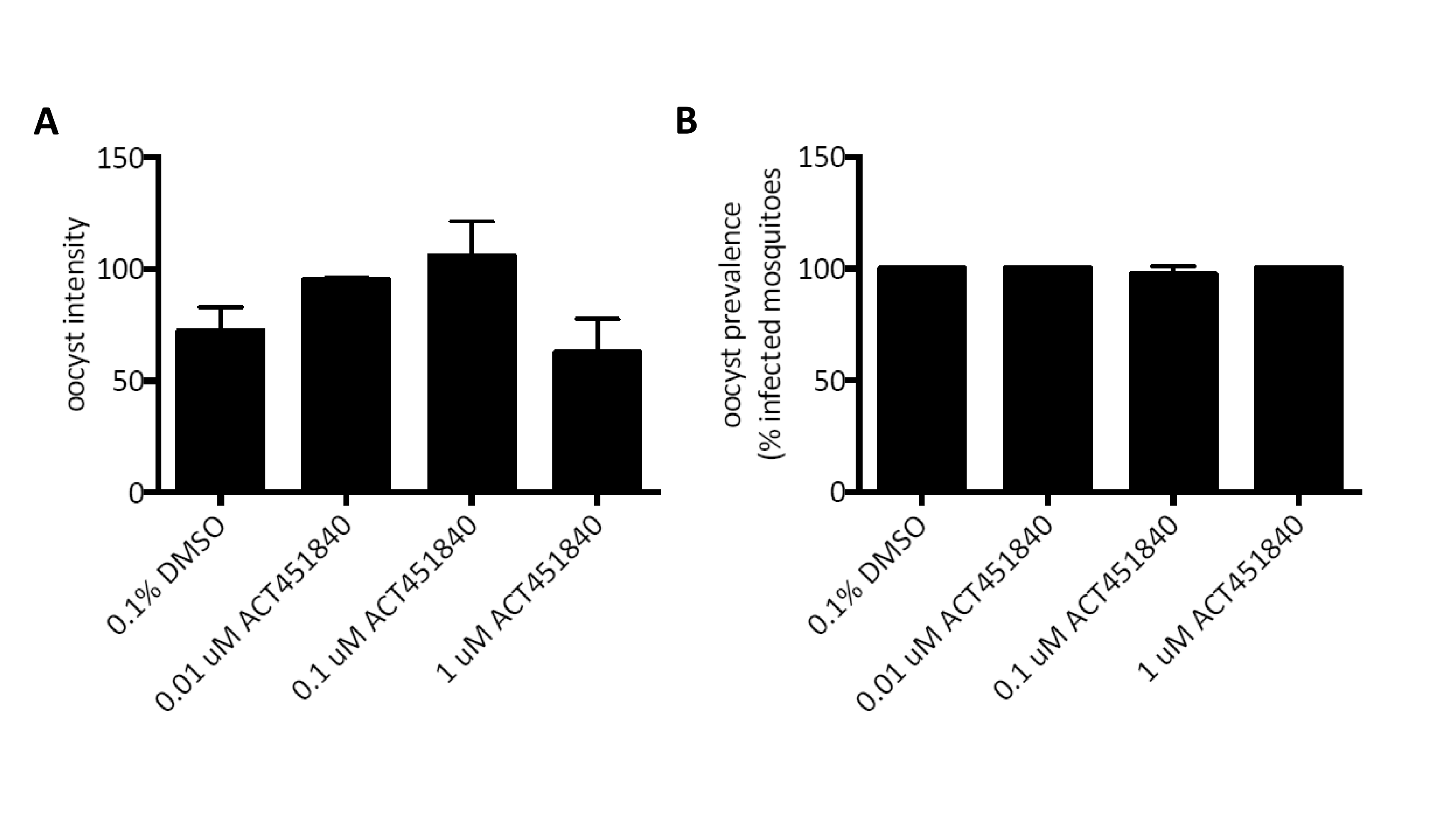

Supplement: S2 Fig — Standard membrane feeding assays were performed without a 24-h preincubation of gametocytes with compound (direct mode). The figure shows average oocyst intensity per mosquito (A) and average oocyst prevalence (percentage mosquitoes with at least one oocyst) (B). Error bars indicate SEM from the measurements of the two groups of the 20 mosquitoes per sample. (TIF) [file pmed.1002138.s003.tif]

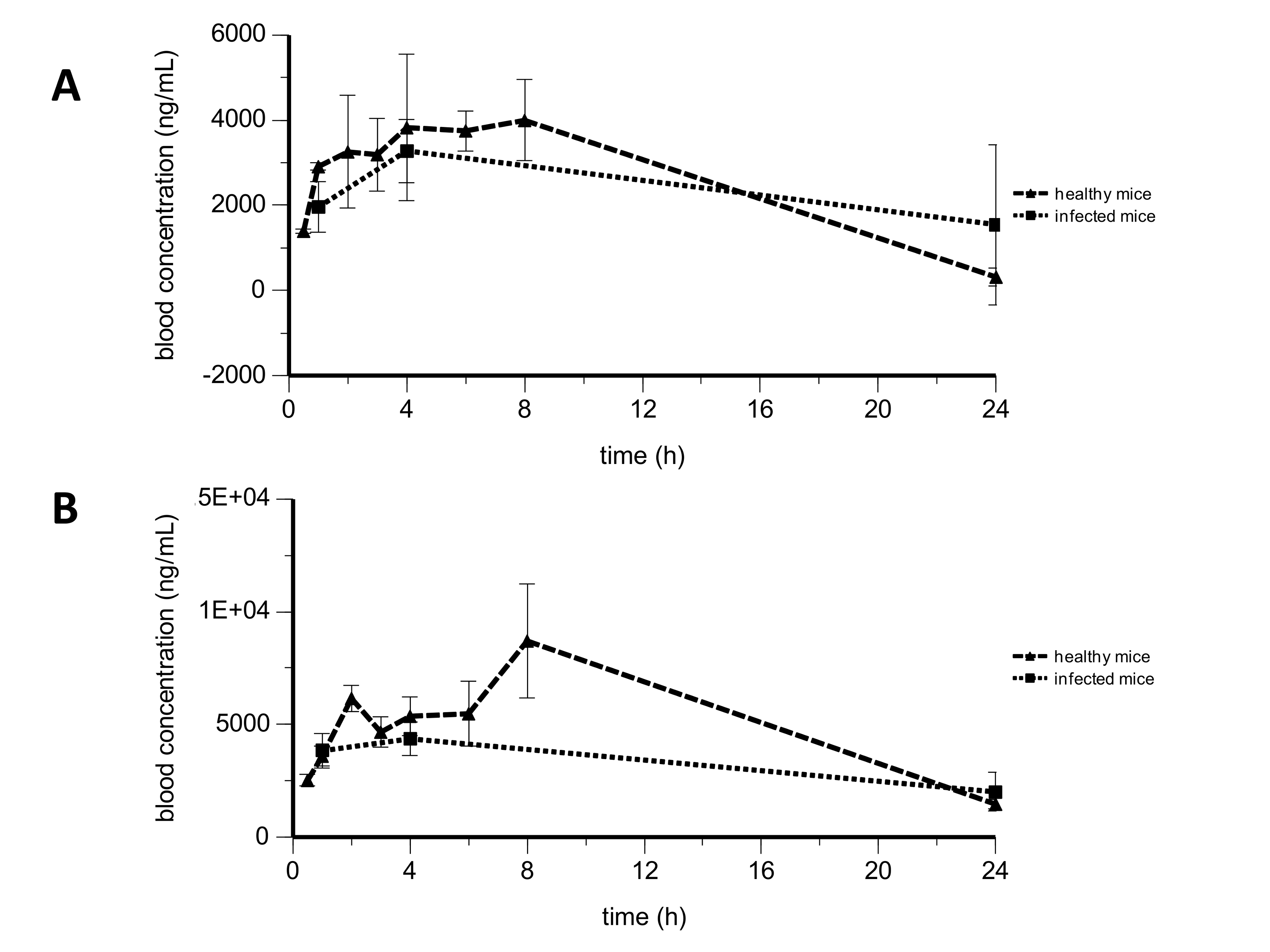

Supplement: S3 Fig — (TIF) [file pmed.1002138.s004.tif]
