# Supplementary material for: Characterization of Novel Antimalarial Compound ACT-451840: Preclinical Assessment of Activity and Dose–Efficacy Modeling
Source: PLoS Med. 2016 Oct 4;13(10):e1002138. doi: 10.1371/journal.pmed.1002138 (PMC5049785; doi:10.1371/journal.pmed.1002138)
Supplement: S1 Text — Antimalarial susceptibility of Cam3.II and V1/S K13 variants. (DOCX) [file pmed.1002138.s005.docx]

***In vitro* antimalarial activity against *P. falciparum* artemisinin resistant strains**

Method:

Parasites were cultured at Columbia University Medical Center (New York, USA) as previously described (1). Cam3.II and V1/S parasites, which had been genetically engineered to express K13 wild-type or K13 C580Y were seeded in a 96-well plate at 0.2% parasitemia and 1% hematocrit (2). Parasites were exposed to a range of ACT-451840, mefloquine, and artesunate drug concentrations for 72 hours. Parasites were then stained with SYBR Green I (Invitrogen) and MitoTracker Deep Red (ThermoFisher Scientific) for 20 minutes at 37^o^C (3). Stained parasites were detected using a BD Accuri 6 Flow Cytometer, and analyzed using FlowJo Software. IC_50_ values were determined by nonlinear regression using GraphPad Prism 6.0. For statistical analyses, Student’s t-tests were performed, and significance was set at *P* < 0.05.

Result:

In 72 hour drug susceptibility assays, the K13 haplotype was found to not alter parasite susceptibility to ACT-451840, mefloquine, or artesunate on both the Cam3.II and V1/S backgrounds. Isogenic parasites that differed only in their K13 haplotype (K13 wild-type vs. K13 C580Y) demonstrated similar IC­­_50_ values that were not significantly different. We note that V1/S parasites were generally more susceptible to ACT-451840, mefloquine, and artesunate compared to Cam3.II parasites. This may in part be due to their differences in PfMDR1 haplotype, as the V1/S strains harbor the N86Y mutation that by gene editing has been shown to increase parasite susceptibility to both mefloquine and the artemisinin metabolite dihydroartemisinin (4). Cam3.II has the Y184F mutation, although that was recently shown to not influence mefloquine or dihydroartemisinin IC_50_ values in gene-edited parasites.

**Antimalarial susceptibility of Cam3.II and V1/S K13 variants.**

|  | ACT-451840 | | | |  | Mefloquine | | | |  | Artesunate | | | |
| --- | --- | --- | --- | --- | --- | --- | --- | --- | --- | --- | --- | --- | --- | --- |
| Parasite | IC_50_  (nM) | IC_50_ fold change | Number of assays | p-value |  | IC_50_  (nM) | IC_50_ fold change | Number of assays | p-value |  | IC_50_  (nM) | IC_50_ fold change | Number of assays | p-value |
| Cam3.II^rev^ | 2.1 ± 0.3 | 1.0 | 5 | - |  | 12.0 ± 1.5 | 1.0 | 5 | - |  | 6.7 ± 0.7 | 1.0 | 5 | - |
| Cam3.II^C580Y^ | 2.3 ± 0.6 | 1.1 | 4 | 0.80 |  | 14.0 ± 0.9 | 1.2 | 5 | 0.30 |  | 6.6 ± 0.3 | 1.0 | 4 | 0.93 |
| V1/S | 0.21 ± 0.001 | 1.0 | 3 | - |  | 1.3 ± 0.4 | 1.0 | 4 | - |  | 1.9 ± 0.03 | 1.0 | 3 | - |
| V1/S^C580Y^ | 0.19 ± 0.015 | 0.9 | 3 | 0.14 |  | 0.8 ± 0.3 | 0.6 | 4 | 0.35 |  | 1.9 ± 0.39 | 1.3 | 3 | 0.99 |

IC50 values were calculated from 72 h dose-response data measured by flow cytometry of parasites stained with SYBR Green and Mitotracker Deep Red. Values indicate mean ± SEM, shown in nM. Significance determined by Student's t-test against parental lines.

**Reference**

1. Fidock D a, Nomura T, Wellems TE. Cycloguanil and its parent compound proguanil demonstrate distinct activities against Plasmodium falciparum malaria parasites transformed with human dihydrofolate reductase. Mol Pharmacol [Internet]. 1998;54(6):1140–7. Available from: http://www.ncbi.nlm.nih.gov/pubmed/9855645

2. Straimer J, Gnädig NF, Witkowski B, Amaratunga C, Duru V, Ramadani AP, et al. K13-propeller mutations confer artemisinin resistance in Plasmodium falciparum clinical isolates. Science (80- ). 2014;2624(1985):428–31.

3. Ekland EH, Schneider J, Fidock D a. Identifying apicoplast-targeting antimalarials using high-throughput compatible approaches. FASEB J [Internet]. 2011;25(10):3583–93. Available from: http://www.pubmedcentral.nih.gov/articlerender.fcgi?artid=3177575&tool=pmcentrez&rendertype=abstract

4. Veiga, M. I., Dhingra, S. K., Henrich, P. H., Straimer, J., Gnadig, N., Uhlemann, A. C., Martin, R. E., Lehane, A. M., and Fidock, D. A. (2016) Globally prevalent PfMDR1 mutations modulate *Plasmodium falciparum* susceptibility to artemisinin-based combination therapies, *Nat Commun* *(in press)*.
