# Supplementary material for: Characterization of Novel Antimalarial Compound ACT-451840: Preclinical Assessment of Activity and Dose–Efficacy Modeling
Source: PLoS Med. 2016 Oct 4;13(10):e1002138. doi: 10.1371/journal.pmed.1002138 (PMC5049785; doi:10.1371/journal.pmed.1002138)
Supplement: S2 Text — (DOCX) [file pmed.1002138.s006.docx]

***In vivo* asexual blood stage activity**

**S2 Tab. *In vivo* efficacy and exposure of ACT-451840 against *P. berghei***

|  | **Efficacy data**  **(measured)** | | | **PK parameters**  **(predicted)** | | | |
| --- | --- | --- | --- | --- | --- | --- | --- |
|  | **Oral dose**  **(mg/kg)** | **Antimalarial activity (%)** | **Survival**  **(days)** | **T_max_**  **(h)** | **C_max_ (ng/mL)** | **Total AUC over the treatment period (ng/mL*h)** | **Time over threshold (h)** |
| Single- dose regimen | 0 | 0.00 | 5.0 |  | 0 | 0 | 0 |
|  | 10 | 21.75 | 5.0 | 3 | 617 | 4170 | 0 |
|  | 15 | 39.00 | 5.0 | 3 | 901 | 6260 | 0 |
|  | 20 | 76.23 | 7.0 | 3 | 1170 | 8350 | 0 |
|  | 25 | 90.29 | 6.7 | 3 | 1420 | 10400 | 0 |
|  | 30 | 97.42 | 7.9 | 3 | 1670 | 12500 | 0 |
|  | 60 | 99.40 | 13.7 | 4 | 2900 | 25100 | 7 |
|  | 80 | 98.88 | 12.7 | 4 | 3540 | 33400 | 9 |
|  | 100 | 99.19 | 12.4 | 6 | 4090 | 41800 | 11 |
|  | 300 | 98.84 | 15.7 | 6.5 | 6690 | 126000 | 24 |
| triple–dose regimen | 3 | 0.00 | 6.0 | 27 | 192 | 3750 | 0 |
|  | 10 | 32.25 | 6.0 | 27 | 617 | 12500 | 0 |
|  | 30 | 99.53 | 13.0 | 51 | 1670 | 37600 | 0 |
|  | 2 x 15^2^ | 99.06 | 9.0 | 35 | 1060 | 37600 | 0 |
|  | 2 x 30^2^ | 99.81 | 14.6 | 59 | 1980 | 75200 | 8 |
|  | 50 | 99.78 | 15.6 | 27 | 2530 | 62700 | 16 |
|  | 100 | 99.84 | 21.8 – 20% cure^1^ | 52 | 4100 | 125000 | 32 |
|  | 300 | 99.78 | 30.0 – 100% cure^1^ | 54 | 6830 | 377000 | 72 |

^1^ No detectable parasites on Day 30 post infection. The 3x 100 and 3x 300mg/kg dosings were performed with n=5 mice.

^2^ The dose is given as the daily dose, once per day except for 2 x 15 mg/kg and 2 x 30 mg/kg where mice were dosed twice per day, the second daily dose 8 h after the first dose.
