# Supplementary material for: Characterization of Novel Antimalarial Compound ACT-451840: Preclinical Assessment of Activity and Dose–Efficacy Modeling
Source: PLoS Med. 2016 Oct 4;13(10):e1002138. doi: 10.1371/journal.pmed.1002138 (PMC5049785; doi:10.1371/journal.pmed.1002138)
Supplement: S3 Text — (DOCX) [file pmed.1002138.s007.docx]

**Blood to plasma ratio**

Method:

The blood to plasma ratio was determined using ^14^C-labeled ACT-451840 at Actelion Pharmaceuticals Ltd. (Allschwil, Switzerland). For this, human fresh whole-blood was spiked with a mixture of non-labeled and ^14^C-labeled ACT-451840 at a nominal concentration of 0.5 μg / mL and incubated for 60 minutes on a rotary mixer at 37 °C. Radioactivity was determined by liquid scintillation counting in whole blood and after preparation of plasma.

Result:

The ratio of the blood to plasma concentration was found to be 0.6.
