# Supplementary material for: Characterization of Novel Antimalarial Compound ACT-451840: Preclinical Assessment of Activity and Dose–Efficacy Modeling
Source: PLoS Med. 2016 Oct 4;13(10):e1002138. doi: 10.1371/journal.pmed.1002138 (PMC5049785; doi:10.1371/journal.pmed.1002138)
Supplement: S4 Text — Pharmacokinetic parameters of ACT-451840 after 100 mg single-dose oral administration of various formulations in dogs. (DOCX) [file pmed.1002138.s008.docx]

**Effect of formulation on pharmacokinetic parameters in dogs**

Method:

The fumarate salt of ACT-451840 was administered orally at 10 mg/kg to fasted and fed male dogs (100 mg/dog, n = 4) as a suspension in an aqueous vehicle containing 0.5% (w/w) methylcellulose. In an additional study, pharmacokinetic parameters in fasted dogs (n = 4, male) were compared after 100 mg oral doses of ACT-451840 formulated as an aqueous suspension (powder of ACT-451840 with mannitol, xanthan gum, and colloidal silica as inactive ingredients reconstituted with water), a solution in corn oil, and a solution in Phosal 53 MCT (phosphatidylcholine solubilized in a carrier system).

Result:

A significant food effect was observed in fed dogs; they had 5.7-fold higher AUC and 3.7-fold higher Cmax values than fasted dogs. The additional pharmacokinetic study was performed to identify a formulation that could mimic the food effect after oral dosing in dogs. Administration of both the corn oil and Phosal 53 MCT solutions resulted in higher plasma exposure than the suspension formulation. For the corn oil and Phosal 53 MCT solutions, AUC increased 7.8- and 5.0-fold and Cmax increased 8.1- and 4.3-fold, respectively.

**S4 Tab**

| **Parameter** | **Formulation** | **Food status** | **Geometric Mean** | **Range** |
| --- | --- | --- | --- | --- |
| AUC_0-∞_  (ng∙h/mL) | Aqueous suspension containing 0.5% methylcellulose | Fasted | 304 | 117 - 743 |
|  | Aqueous suspension containing 0.5% methylcellulose | Fed | 1720 | 1350 - 2060 |
|  | Aqueous suspension | Fasted | 120 | 33.7 - 234 |
|  | Phosal 53 MCT | Fasted | 597 | 83 - 2760 |
|  | Corn oil | Fasted | 935 | 521 - 1380 |
| C_max_  (ng/mL) | Aqueous suspension containing 0.5% methylcellulose | Fasted | 46.2 | 25.1 - 72.7 |
|  | Aqueous suspension containing 0.5% methylcellulose | Fed | 171 | 122 - 277 |
|  | Aqueous suspension | Fasted | 26.2 | 9.37 - 43.4 |
|  | Phosal 53 MCT | Fasted | 112 | 27 - 440 |
|  | Corn oil | Fasted | 213 | 109 - 301 |

Abbreviations: Area under the plasma concentration versus time curve (AUC) and maximum observed plasma concentration (C_max_).
